# Natural history of artificial urinary sphincter cuff erosion in males: Experience from two tertiary centres in the United Kingdom

**DOI:** 10.1002/bco2.70277

**Published:** 2026-10-09

**Authors:** Loufopoulos Ioannis, Pace Keith, Toia Bogdan, Kapriniotis Konstantinos, Papaefstathiou Efstathios, Newman Thomas, Barratt Rachel, Nobrega Richard, Noah Anthony, Gresty Helena, Sharma Davendra, Greenwell Tamsin, Seth Jai, Ockrim Jeremy

**Affiliations:** ^1^ Functional, Adolescent and Restorative Urology University College Hospital London London UK; ^2^ Urology Department St George's University Hospitals London UK

**Keywords:** artificial urinary sphincter, AUS re‐implantation, incontinence, urethral cuff erosion, urethral stricture

## Abstract

**Objectives:**

This study aimed to describe the natural history of urethral cuff erosion (UCE) following artificial urinary sphincter (AUS) implantation, including the incidence of urethral stricture and fistula formation, and the outcomes of salvage AUS reimplantation, based on the experience of two UK tertiary centres.

**Material and methods:**

Data collected prospectively from 2016 to 2024 was analysed retrospectively. Demographics, characteristics and outcomes of AUS re‐implantation were analysed.

**Results:**

A total of 273 AUS implantation were performed during the study period. Fifty had device explantation for UCE. Median age at explantation was 73 years. Median time from implantation to erosion was 32 months. About 12% had a history of neurogenic bladder and 58% pelvic irradiation. The most significant cause of acute erosion was traumatic catheterisation (44%).

Following explantation, nine developed urethral stricture (18%), while five patients had urethro‐cutaneous fistulae (10%). Twelve individuals underwent salvage AUS re‐implantation with 6 (50%) requiring further explantation (50%) within 8 months. Of the remaining 38 patients, 17 continued with pad use alone and 21 underwent alternative conservative management including long‐term catheterisation, convene drainage, penile clamp or urethral bulking. Four patients (8%) ultimately required urinary diversion, two following primary explantation and two following failed salvage reimplantation.

**Conclusion:**

Overall, 18% of males having AUS implantation had UCE and explantation of the device. The vast majority of them had a history of pelvic irradiation or catheterisation for stricture disease (76%). The main risk for UCE was traumatic catheterisation. About 18% of explanted cases developed urethral stricture and 10% urethra‐cutaneous fistulae. Only 24% of these patients had attempted salvage AUS re‐implantation, with one half resulting in further early UCE/infection and removal.

## 1 INTRODUCTION

Stress urinary incontinence (SUI) remains a prevalent and bothersome condition affecting a significant proportion of men following radical prostatectomy and other pelvic interventions. For decades, the artificial urinary sphincter (AUS) has been the gold standard surgical treatment for moderate to severe male SUI, offering high rates of patient satisfaction and durable continence outcomes.^1^, ^2^ The estimated revision free survival rate is 50%–70% in 10 years.^1^, ^3^, ^4^ Despite good durability, AUS implantation is not without complications. Mechanical failure, urethral atrophy, cuff erosion and infection are recognised adverse events that can significantly impact device function and patient quality of life.^5^ Among these, cuff erosion, through the urethral mucosa, is one of the most serious, requiring urgent early explantation and complex downstream management. The estimated rates of device erosion are highly variable in the literature, with data describing rates between 0.46% and 27.8%.^3^, ^4^, ^5^, ^6^, ^7^, ^8^, ^9^, ^10^, ^11^, ^12^

Cuff erosion may present insidiously with recurrent incontinence, haematuria or perineal pain, but it can also manifest acutely with infection or urethral necrosis. The aetiology of erosion is likely multifactorial, involving chronic pressure ischemia at the cuff–urethral interface, impaired healing in irradiated or previously operated tissues and often contributions from repeated urethral instrumentation and superimposed infection. Additional risk factors have been identified, including medical factors such as coronary artery disease (CAD), hypertension and neurogenic bladder; previous urological interventions especially revision surgeries and previous urethroplasty; and technical factors especially smaller cuff sizes.^2^, ^3^, ^9^ Of note, one of the most established causes of AUS erosion is traumatic catheterisation with cuff inflated, usually occurring in primary care or in emergency settings.^9^

Urgent explantation of the device is necessary to avoid sepsis and preserve urethral integrity. Primary urethral reconstruction at the time of explantation is recommended where feasible, as failure to repair the urethral defect risks ongoing extravasation and local infection, which may significantly compromise subsequent re‐implantation. Among the complications, urethral strictures demonstrate a high prevalence of 10%–40%.^9^, ^13^, ^14^, ^15^ Concurrent infection and localised inflammation and ischaemia, subsequent fibrosis has been proposed as the underlying mechanisms of urethral stricture formation. Higher severity of erosion, circumferential erosions and previous history of pelvic irradiation have been shown to correlate with higher rates of strictures compared to partial, simple and noninfected cases.^13^, ^14^ The surgical technique of repair of the erosion does not seem to affect the stricture rates.^13^ Management requires prolonged catheterisation, need for reconstructive urethral surgery or urinary diversion.

Furthermore, the decision to pursue salvage AUS implantation after explantation must be weighed carefully. Salvage AUS refers to complete device reimplantation rather than isolated cuff replacement. In both our centres, all AUS implantations—primary and salvage/second—are performed via a perineal approach and a simultaneous groin incision, with salvage reimplantation sited at the same or more proximal urethral location depending on underlying tissue condition. While salvage AUS offers the possibility of restored continence, it carries a higher risk of re‐erosion/infection and less functional efficacy compared to primary implants.^4^, ^12^, ^13^ Surgical modifications, such as transcorporeal cuff placement interposition grafts or staged implantation, have been proposed to mitigate these risks, but comparative data remain limited.

Although the MASTER study gave level 1 data of UK practice and device efficacy,^16^ national‐level data on AUS cuff erosion remain sparse, and no large‐scale multicentre studies have explored the outcomes of erosion, stricture development or salvage AUS performance in British patients. This paper addresses this gap in knowledge.

## 2 MATERIAL AND METHODS

This study is a retrospective cohort analysis of male patients who underwent AUS cuff explantation for confirmed urethral erosion at two UK tertiary referral centres in a 10‐year period between January 2015 and September 2024. Both institutional surgical databases were screened to identify patients who had AUS insertion and subsequently underwent device explantation. Urethral cuff erosion (UCE)—defined as erosion of the urethral wall at the cuff site with mucosal breach and direct exposure of the device to the urinary tract—was confirmed intraoperatively via cystoscopy. Cross‐sectional imaging (CT or MRI) was performed in all cases to assess for periprosthetic fluid collections or abscess formation and evaluate the extent of surrounding soft tissue infection, prior to surgical planning. Patients undergoing AUS explantation for other indications, such as mechanical failure without erosion, were excluded.

Demographic data, clinical history, intraoperative findings and postexplant outcomes were obtained from electronic medical records. Extracted variables included patient age, body mass index (BMI), comorbidities (including diabetes, hypertension, cardiac disease and smoking history) and history of pelvic radiotherapy. AUS‐specific data included cuff size, location, surgical approach (standard or transcorporal), number of prior AUS procedures and time intervals between initial AUS placement, erosion‐explantation and any subsequent reimplantation. The aetiology and severity of erosion were recorded where available, as were intraoperative management strategies during explantation.

Postoperative outcomes included the development of urethral stricture, fistula, infection and need for further reconstructive procedures or urinary diversion. For patients undergoing salvage AUS reimplantation, subsequent complications such as re‐erosion, infection and continence were also recorded. Median follow‐up time was calculated from the date of explantation to the most recent urology clinic visit or surgical event.

Continuous variables are reported as medians with interquartile ranges (IQR) and categorical variables as frequencies and percentages. Formal hypothesis testing and multivariable regression modelling were not undertaken, as the number of events within key subgroups (e.g., urethral stricture and re‐erosion) was insufficient to allow for robust statistical comparisons without risk of overfitting or type II error.

## 3 RESULTS

A total of 273 AUS implants were performed in this time period. A total of 50 patients underwent AUS cuff explantation due to urethral erosion during the study period and were included in the analysis, representing 18% of the total cohort having an AUS implantation. The median age at the time of AUS implantation was 70 years (IQR 10), and median age at the time of erosion was 73 years (IQR 11). The median BMI was 27.5 kg/m^2^ (IQR 7).

The median time from primary AUS implantation to the development of cuff erosion was 32 months (IQR 64). In 24 cases (48%), erosion involved less than 50% of the urethral circumference, whereas in 26 cases (52%), it exceeded 50%. Traumatic urethral catheterisation was the most frequently identified contributing factor, reported in 22 patients (44%). Infection‐related erosion was noted in 14 cases (28%), while no specific cause could be determined in 10 patients (20%).

Regarding comorbidity profiles, 29 patients (58%) had a history of pelvic radiotherapy, 14 (28%) had diabetes mellitus and 29 (58%) had essential hypertension. CAD was present in 12 patients (24%) and erectile dysfunction in 16 (32%). Urethral stricture disease prior to AUS implantation was observed in nine patients (18%), and six (12%) had neurogenic bladder. A smoking history was recorded in 30 patients (60%), including 11 current smokers (22%) and 19 former smokers (38%). One patient (2%) had received a previous urethral bulking agent injection. High‐grade prostate cancer (Gleason score ≥8) was noted in eight patients (16%), with the vast majority (88%) having the AUS implanted due to postradical prostatectomy SUI. Most devices were implanted at the bulbar urethra (78%), with cuff sizes ranging from 3.5 to 6.0 cm. The most commonly used cuff size was 4.5 cm (32%), followed by 4.0 cm (26%). A 3.5‐cm cuff, which may be associated with higher risk of erosion in atrophic urethras, was used in six patients (12%). Transcorporal cuff placement was performed in only one patient (2%). Findings are summarised in Table 1 and Figures 1 and 2.

**TABLE 1:** Patient demographics.

	Median	IQR
Age at implantation (years)	70	10
Age at removal (years)	73	11
BMI (kg/m^2^)	27.5	7
	*N*	%
Smoking/former smoker	11/19	22/38
Neurogenic bladder	6	12
Urethral stricture	9	18
Pelvic irradiation	29	58
Erectile dysfunction	16	32
Hypertension	29	58
Diabetes	14	28
Coronary artery disease	12	24
Previous bulking urethral injection	1	2
Gleason score ≥8 prostate cancer (high grade)	8	16
Position of cuff		
Bladder neck	3	6
Bulbar urethra	39	78
Proximal urethra	1	2
Trans‐corporeal	1	2
Not documented	5	10
Size of cuff (cm)		
3.5	6	12
4	13	26
4.5	16	32
5	1	2
6	1	2
Not documented	13	26

**FIGURE 1:**
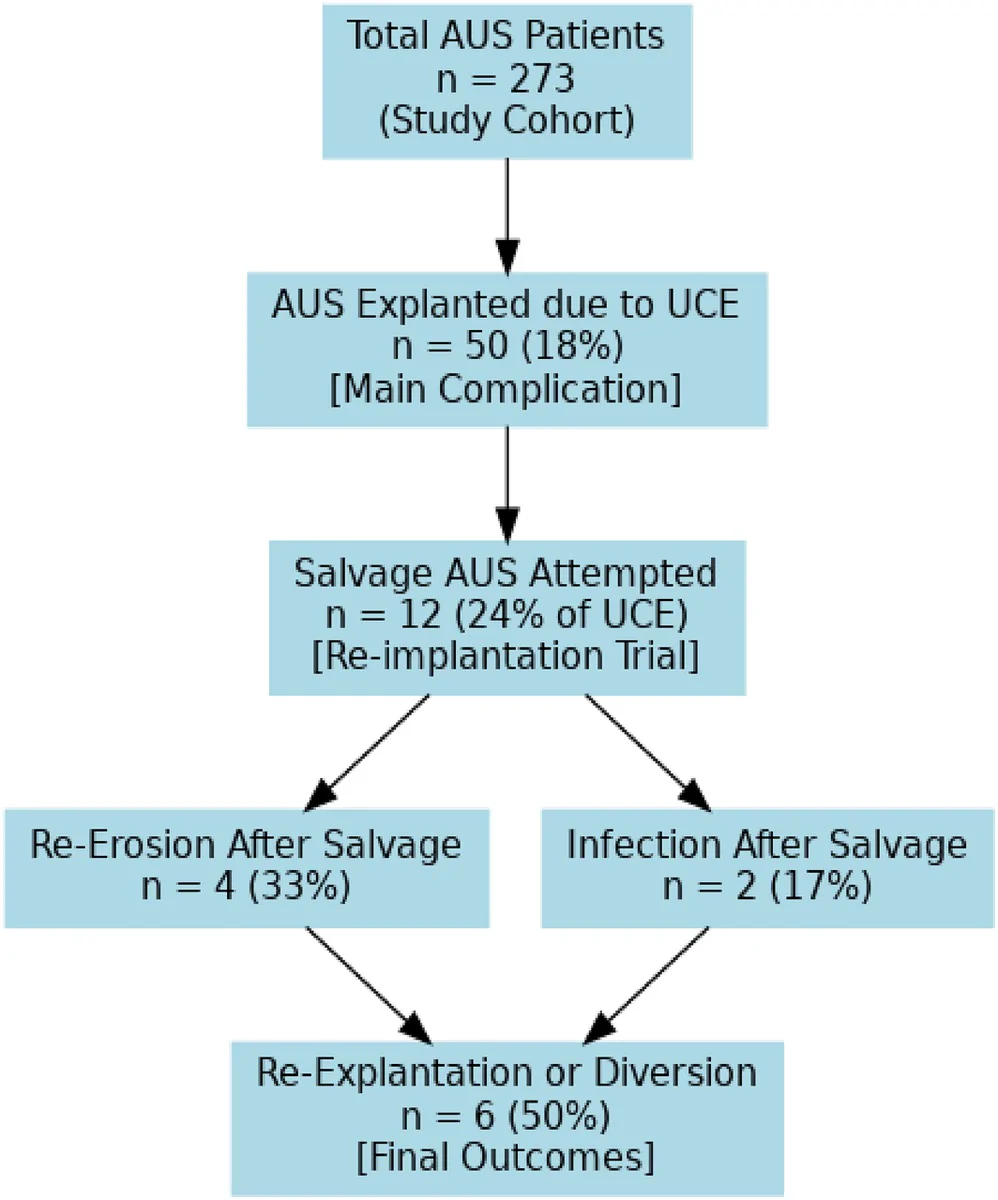
Flow chart: outcomes following artificial urinary sphincter (AUS) explantation for urethral cuff erosion.

**FIGURE 2:**
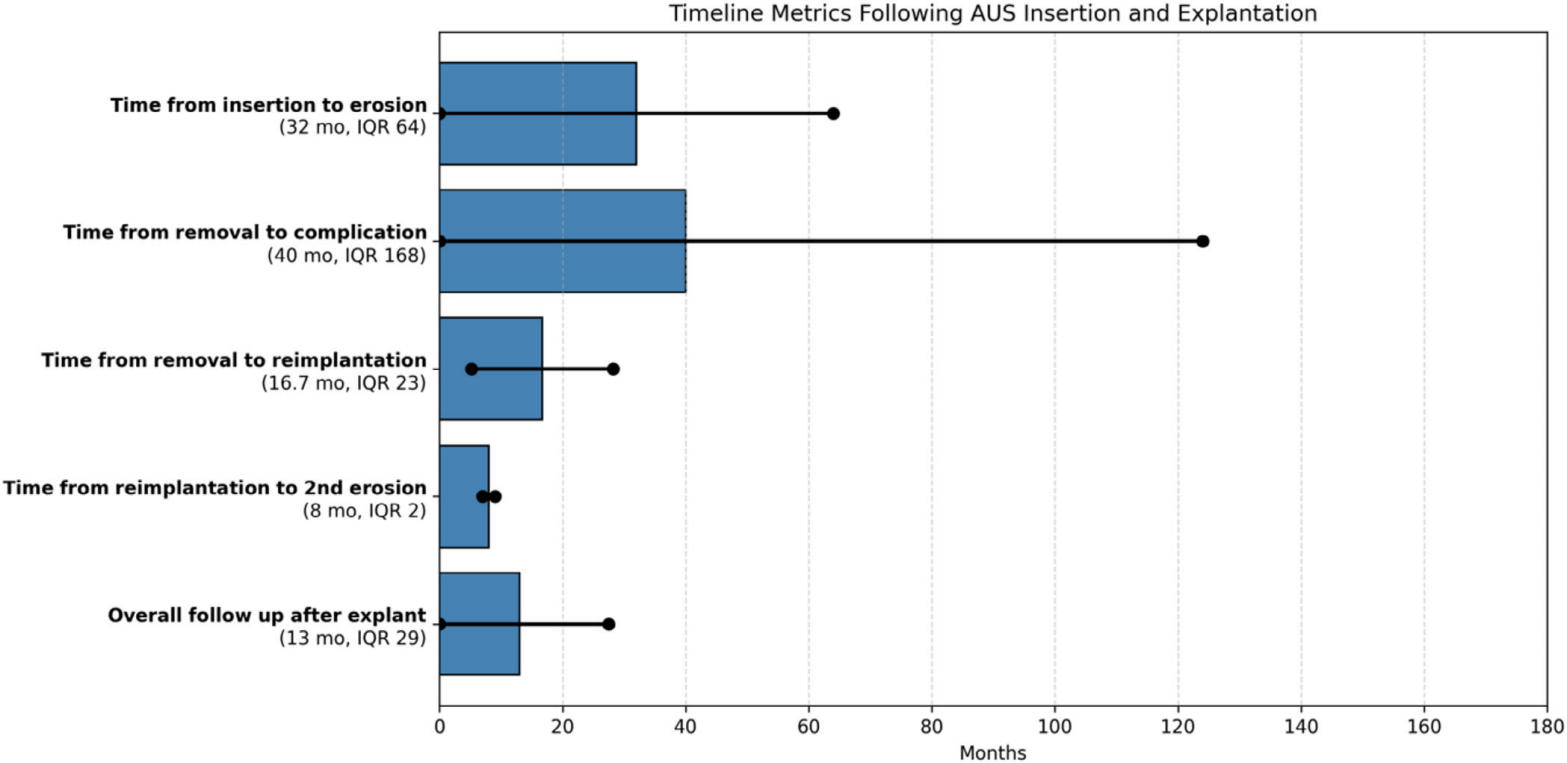
Median timeline intervals following AUS implantation, erosion, explantation and salvage procedures.

At the time of AUS explantation, intraoperative management varied depending on the extent of urethral injury. Twenty‐four patients (48%) were managed conservatively with urethral catheter drainage alone, whereas primary buccal mucosa urethroplasty reconstruction was undertaken in 20 patients (40%), and a primary urethral anastomosis was performed in six cases (12%). Postexplantation complications were observed in 30% of patients. Urethral stricture occurred in nine patients (18%), while urethrocutaneous fistula formation was noted in five patients (10%) of whom four had undergone urethroplasty. One patient (2%) died due to sepsis following AUS erosion. The remaining 70% of patients did not experience further urological complications following explantation. The median time from AUS explantation to the development of a documented complication was 40 months (IQR 168) (see Figure 2).

Of the 50 patients, 12 (24%) underwent salvage AUS reimplantation following a variable period of urethral healing. The median time between explantation and salvage reimplantation was 16.7 months (IQR 23). In the majority of cases, salvage reimplantation was performed at the same urethral location, based on intraoperative assessment of tissue quality and urethral integrity.

Among the salvage cohort, four patients (33%) experienced re‐erosion of the second device with no infection, with a median time to re‐erosion of 8 months (IQR 2), while early postoperative infection of the salvage AUS occurred in two patients (17%). Six of the 12 re‐implant patients (50%) ultimately required re‐explantation (see Table 2).

**TABLE 2:** Natural history of AUS cuff erosion.

Cause of erosion		
Infected device	14	28
Traumatic catheterisation	22	44
No obvious cause	10	20
Not documented	4	8
Severity of erosion (% of circumference of urethra)		
<50%	24	48
>50%	26	52
Intra‐operative technique of repair of erosion		
Catheter only	24	48
Urethral reconstruction	20	40
Primary urethral anastomosis	6	12
Complication postexplantation		
Urethral stricture	9	18
Urethro‐cutaneous fistula	5	10
Septic death	1	2
AUS re‐implantation	12/50	24
Complication of re‐implanted device		
Infected‐eroded device	2/12	16.7
UCE	4/12	33.3
Explantation of new device	6/12	50

Overall, 38 patients (76%) were managed without further continence surgery. Of these, 17 continued with pad use alone, while 21 underwent alternative conservative management including long‐term catheterisation, convene drainage, penile clamp or urethral bulking (without success). A total of four patients (8%) ultimately required urinary diversion via suprapubic catheterisation or ileal conduit formation, two following primary explantation and two following failed salvage reimplantation. The median overall follow‐up duration after AUS explantation was 13 months (IQR 29) (Figure 2).

## 4 DISCUSSION

Much of the current literature on AUS erosion and salvage management originates from North American centres, with limited data available from UK practice. In 2024, Findlay et al. reported one of the most comprehensive studies on this topic to date, analysing outcomes of 105 patients with cuff erosion across multiple US institutions.^14^ Their findings highlighted a 32% postexplant stricture rate and a 37% re‐erosion rate after salvage AUS. We note that healthcare systems, patient demographics and surgical approaches may differ between the United States and United Kingdom, limiting generalisability.

Cuff erosion is reported to occur in 0.46%–27.8% of patients in the literature,^3^, ^4^, ^5^, ^6^, ^7^, ^8^, ^9^, ^10^, ^11^, ^12^ with the largest European multicentre series, the DOMINO (Debates on Male Incontinence) group, reporting an overall urethral erosion rate of 12.8% across 473 patients in 16 central European centres.^17^ The 18% figure reported in this cohort lies within this range and is likely explained by the substantially higher proportion of previously irradiated patients in our cohort (58% vs 29.5% in the DOMINO series^17^), as previous pelvic radiation therapy and a history of urethroplasty have been shown to be independent risk factors for earlier time to erosion.^14^, ^18^ The DOMINO group identified prior irradiation as carrying a hazard ratio of 3.38 for erosion, underscoring its dominant influence as a risk factor for UCE. Of note, both our centres use an exclusively perineal approach, which the DOMINO data suggest is otherwise protective compared to the penoscrotal route.^17^

The predominant cause for UCE in this cohort was traumatic catheterisation at 44%; this is in keeping with other studies which have reported rates to be 26% and 54%,^9^, ^19^ and emphasises the need for improved prevention strategies. Different approaches have been suggested to help reduce the rate of UCE from traumatic catheterisation including patient education and the use of medical alert bracelets, as well as continued education of clinical (especially) accident and emergency staff.^20^ The British Association of Urological Surgeons (BAUS) and Nurses (BAUN) consensus document on the management of the complications of long‐term indwelling catheters also gives instructions on troubleshooting problems with long‐term indwelling catheters including in patients with AUS.^21^

There are data to support that a preoperative history of diabetes mellitus, smoking, peripheral vascular disease and the use of androgen deprivation therapy also increase rates of cuff erosion, with diabetes being independently associated with a 2.3‐fold increased risk of AUS erosion‐infection.^22^ About 28% of patients in our study had a history of diabetes mellitus; this is significantly higher than the total type 2 diabetes prevalence in England, which was estimated at 7.8% for those aged 16 and over in 2021.^23^ This is highly suggestive of a significant correlation and risk factor for erosion and re‐erosion.

Six percent of the cuff erosions reported in this cohort were from sphincters inserted at the bladder neck, while 80% were bulbar urethral artificial sphincters. Current opinion favours a bladder neck insertion for patients who are wheelchair bound, as there is a risk of a perineal pressure sore in this population.^24^ This approach is technically more challenging. However, it has been shown to have a lower AUS erosion rate of 11% compared to a bulbar urethral position rate of up to 27%, while the rates of bulbar erosion in a single cohort of patients with neurogenic stress incontinence with a background of spina bifida were reported as 15%.^24^ However, neurogenic incontinence represents a distinct clinical entity, with unique characteristics including the underlying neurological condition and mode of bladder evacuation, which may significantly influence erosion rates and outcomes; therefore, these figures should not be directly compared to the nonneurogenic population described in this series.

In our cohort, most cuff erosions happened in patients with cuff sizes of 4 and 4.5 cm. These are the sizes which are most frequently implanted in our centres and are probably the reason for the higher number of erosions in this group. However, literature has reported that the use of a 3.5‐cm cuff is a predictor for cuff erosion^25^ and an independent risk factor for mechanical failure.^26^ It is not clear whether this is related to patient factors requiring a smaller cuff placement or device‐related technical specifications (including higher micro fracture rates of the tighter cuff).^26^ Notably, more than half of our cohort presented with urethral erosion involving over 50% of the urethral circumference, highlighting the extent of tissue compromise at the time of diagnosis.

Management of urethral erosion is immediate removal of the AUS device (cuff, pump and pressure‐regulating balloon) and subsequent urinary catheter placement,^27^ and this was done in 48% of cases in this cohort, whereas the others underwent primary urethral reconstruction when it was deemed possible by the emergency clinician. Overall, stricture rates after AUS explant for UCE 8.3% and 32%^13^, ^28^ which is in keeping with the 18% who developed urethral strictures in this cohort. In situ urethroplasty at the time of explant has been reported to improve urethral stricture rates post operatively in some series.^29^ Caldwell et al. showed in a series of 37 men managed with intraoperative urethroplasty, 35% developed urethral strictures, compared with 85% of those treated with catheter alone at 12 months. However, our experience is that stricture rate was 25% (6/24 patients) with catheter drainage alone versus 12% (3/26 patients) with primary urethroplasty. No demographic or intraoperative variables associated with the eventual development of a urethral stricture have been recognised to date.^30^ Our cohort also has a 10% urethro‐cutaneous fistulae rate; this is in keeping with the 6.1% rates reported by Loh‐Doyle et al.^31^ Notably, 80% of fistulae in our cohort occurred in patients who underwent primary urethroplasty at the time of explantation. This is not unexpected, as urethral erosion is by definition an infected field, and urethroplasty performed in this context carries an inherently higher risk of fistula formation due to the compromised tissue quality and bacterial contamination at the erosion site.

Only 24% of the patients in this cohort had an attempted salvage AUS re‐implantation, with the rest opting for conservative options or diversion. The median time to salvage reimplantation was 16.7 months, longer than the typically recommended 3–6 months, which likely reflects the significant disruption to elective surgical services during the COVID‐19 pandemic throughout the study period. About 50% of reimplantation resulted in further early UCE or infection and removal. This is also in keeping with other studies which have shown that a history of UCE is the biggest risk factor for another UCE after reimplantation.^25^, ^31^ These findings underscore the long‐term functional burden associated with AUS erosion and explantation and reinforce the importance of comprehensive preoperative counselling regarding continence prognosis and quality‐of‐life outcomes following device removal.

While alternative salvage strategies including transcorporal cuff placement, tandem cuff configuration, cuff downsizing and staged reimplantation have been described in the literature, their use in our centres is selective. Transcorporal cuff placement was employed in only one patient (2%) in this cohort, reflecting its use in cases of severe urethral compromise, urethral atrophy or after previous erosion where standard cuff placement is no longer feasible. Guralnick et al.^32^ first described the transcorporal technique in 31 patients requiring revision for erosion or urethral atrophy, reporting an 84% continence rate with no erosion or infection of transcorporally placed cuffs, though noting a potential risk of erectile function deterioration in potent men. A recent systematic review and meta‐analysis^33^ confirmed a significantly higher revision rate with transcorporal placement compared to standard cuff placement (OR 2.99, 95% CI 1.16–7.75), while demonstrating significantly lower erosion (OR 0.35, 95% CI 0.15–0.81) and infection rates (OR 0.33, 95% CI 0.12–0.95), with comparable continence and quality of life outcomes. Cuff downsizing may also be considered intraoperatively where urethral atrophy is identified at the time of revision. The decision between salvage reimplantation, conservative management and urinary diversion in our centres is made through structured multidisciplinary team (MDT) discussion, incorporating patient expectations, comorbidity profile, tissue quality and quality‐of‐life priorities.

Urinary diversion was required in a minority of patients in this cohort (8%), reserved as a definitive management strategy for the most complex cases including multiple erosions, multiple failed AUS procedures, extensive urethral fibrosis, urethral necrosis or significant patient frailty. Two patients underwent suprapubic catheterisation or ileal conduit formation following primary explantation and a further two following failed salvage reimplantation. All patients who underwent urinary diversion reported satisfaction with their quality of life at follow‐up, suggesting that in appropriately selected and counselled patients, urinary diversion can represent an acceptable and effective long‐term solution.

In terms of our study's limitations, we acknowledge its retrospective design, relatively small sample size and the absence of a comparator group as key sources of bias. As the dataset was compiled from two tertiary referral centres, there may be referral bias toward more complex cases. For this reason, only descriptive analyses were performed, with results presented as medians and proportions. The small number of events within key subgroups (e.g., re‐erosion, stricture and fistula) precluded reliable multivariable analysis without risking overfitting or misleading associations. Despite these constraints, this cohort remains the largest reported UK series specifically examining the natural history of AUS erosion and outcomes following explantation.

## 5 CONCLUSIONS

AUS cuff erosion remains a significant complication following device implantation, often associated with substantial downstream morbidity including urethral stricture, fistula and recurrent incontinence. Our two‐centre experience demonstrates that traumatic catheterisation is the most common identifiable cause and that salvage AUS reimplantation carries a high risk of further erosion–infection in up to 50%. Notably, over half of patients in this cohort ultimately decided upon alternative long‐term continence strategies. These findings highlight the need for improved prevention, early recognition of erosion to try and reduce urethral loss and careful patient selection and counselling regarding both initial implantation and management following erosion. Further prospective studies are needed to evaluate optimal surgical reconstruction strategies to improve the long‐term functional outcomes in this high‐risk group.

## AUTHOR CONTRIBUTIONS

**Ioannis Loufopoulos, Keith Pace:** Conceptualization; methodology; data curation; formal analysis; investigation; visualisation; writing. **Bogdan Toia, Konstantinos Kaprioniotis, Efstathios Papaefstathiou, Thomas Newman, Rachel Barratt, Richard Nobrega, Anthony Noah, Helena Gresty, Davendra Sharma:** Data curation and manuscript review. **Tamsin Greenwell, Jai Seth:** Supervision; methodology; and critical revision of the manuscript. **Jeremy Ockrim:** Conceptualization; supervision; methodology; and critical revision of the manuscript.

## CONFLICT OF INTEREST STATEMENT

The authors declare no conflicts of interest.

## PATIENT CONSENT STATEMENT

Patient consent was not required for this study due to its retrospective design using anonymised data.

## PERMISSION TO REPRODUCE MATERIAL

No previously published material, figures or tables were reproduced in this manuscript.

## Data Availability

The data that support the findings of this study are available from the corresponding author upon request. The data are not publicly available due to institutional data protection policies.
